# Low P16^INK4A^ Expression Associated with High Expression of Cancer Stem Cell Markers Predicts Poor Prognosis in Cervical Cancer after Radiotherapy

**DOI:** 10.3390/ijms19092541

**Published:** 2018-08-27

**Authors:** Hung-Chun Fu, I-Chieh Chuang, Yi-Chien Yang, Pei-Chin Chuang, Hao Lin, Yu-Che Ou, Chan-Chao Chang Chien, Hui-Shan Huang, Hong-Yo Kang

**Affiliations:** 1 Graduate Institute of Clinical Medical Sciences, College of Medicine, Chang Gung University, Kaohsiung 833, Taiwan; allen133@cgmh.org.tw (H.-C.F.); yichienyang@gmail.com (Y.-C.Y.); 2 Center for Menopause and Reproductive Medicine Research, Kaohsiung Chang Gung Memorial Hospital, Kaohsiung 833, Taiwan; 3 Department of Obstetrics and Gynecology, Kaohsiung Chang Gung Memorial Hospital and Chang Gung University College of Medicine, Kaohsiung 833, Taiwan; haolin@cgmh.org.tw (H.L.); ou4727@cgmh.org.tw (Y.-C.O.); 17377@ms17.hinet.net (C.-C.C.C.); 4 Department of Anatomic Pathology, Kaohsiung Chang Gung Memorial Hospital and Chang Gung University College of Medicine, Kaohsiung 833, Taiwan; b9205043@cgmh.org.tw (I-C.C.); maggie1211@cgmh.org.tw (H.-S.H.); 5 Department of Dermatology, Kaohsiung Chang Gung Memorial Hospital and Chang Gung University College of Medicine, Kaohsiung 833, Taiwan; 6 Stem Cell Research Core Laboratory, Department of Medical Research, Kaohsiung Chang Gung Memorial Hospital, Kaohsiung 833, Taiwan; jessica_4031976@yahoo.com.tw

**Keywords:** P16^INK4A^, SOX2, ALDH1A1, radioresistance, cervical cancer, cancer stem cells

## Abstract

Previous studies have suggested that cancer stem cells (CSCs) resisted radiotherapy and chemotherapy. P16^INK4A^ is a biomarker for cervical carcinogenesis and reduces proliferation of stem cells. We aimed to investigate the expression and clinical significance of cyclin-dependent kinase inhibitor 2A (P16^INK4A^), sex determining region Y-box 2 (SOX2), and Aldehyde dehydrogenase 1 family, member A1 (ALDH1A1) in cervical cancer treated with radiotherapy and cervical cell line models. The expressions of P16^INK4A^, SOX2, and ALDH1A1 were performed by immunohistochemical staining of tumor samples from 139 cervical cancer patients with International Federation of Gynecology and Obstetrics stages Ib to IV. The staining showed high expression in 100, 107, and 13 patients with P16^INK4A^ (>80%), SOX2 (≥10%), and ALDH1A1 (50%), respectively. The high-P16^INK4A^ group had a higher five-year overall survival (OS) rate and disease-free survival (DFS) than the low-P16^INK4A^ group (OS: 62.0% and 35.2%, *p* = 0.016; DFS: 60.0% and 31.2%, *p* = 0.002). The low-P16^INK4A^/high-SOX2 and low-P16^INK4A^/high-ALDH1A1 groups had a worse five-year OS and DFS rate than the high-P16^INK4A^/low-SOX2 and high-P16^INK4A^/low-ALDH1A1 groups, respectively. Depletion of P16^INK4A^ promoted chemoresistance and radioresistance of cervical cancer cells increased the expression of SOX2 and ALDH1A1 and exhibited higher self-renewal ability. These results suggest that lower P16^INK4A^ expression associated with higher CSC markers predicts poor prognostic outcomes and is a promising target in patients with cervical cancer.

## 1. Introduction

Cervical carcinoma is the third most common malignancy diagnosed in women worldwide [1]. Treatments of patients with early-stage (i.e., International Federation of Gynecology and Obstetrics (FIGO) stages IA–IB1) cervical cancer are radical hysterectomy and lymph node dissection. Radiation with or without chemotherapy is conducted for patients with intermediate or high risk of recurrence. Most patients with advanced cervical cancer receive radiation therapy with or without cisplatin-containing chemotherapy as definitive treatments [2,3]. Resistance to radiotherapy is widely recognized as one of the major factors that limit therapeutic efficacy and influence patient outcomes. Ability of radiation resistance in cancer cells is acquired by intrinsic and extrinsic factors. Furthermore, the expression of vascular endothelial growth factor (VEGF), Galectin-1, Sphingosine kinase 1, SKP2, P16^INK4A^, hypoxia, and cancer stem cells (CSCs) may have a major influence on the survival of patients treated with definitive radiotherapy [4,5,6,7,8,9]. However, the detailed cellular or molecular mechanism of the contribution to radiation resistance is still largely unknown.

CSCs are a small subpopulation of tumor cells that have characteristics of tumorigenesis, multilineage differentiation potential, and self-renewal [10]. Due to their self-renewal and tumor-initiating characteristics, CSCs are considered to be the starting point for cancer and may play key roles in cancer relapse and metastasis [11]. Most investigators identify CSCs with stem cell markers for both in vitro and in vivo studies. Additionally, the markers of cervical cancer stem cells (CCSCs) have been accumulating including CD133, CD44, sex determining region Y-box 2 (SOX2), Aldehyde dehydrogenase 1 (ALDH 1), and so on [12]. Previous studies have shown CCSCs were resistant to cisplatin-based chemotherapy and radiotherapy [13,14]. Such cells are proposed to persist in tumor during treatments and cause relapse or metastasis by producing new tumors. Cell-cycle inhibitors hierarchically organize and intrinsically limit CSCs repopulation [15].

P16^INK4A^ is encoded by the CDKN2A gene and is thought to be a tumor suppressor. It is one of the cell-cycle inhibitors and inactivates the cyclin d-cyclin-dependent kinases (CDK) 4/6 complex. P16^INK4A^ prevents the phosphorylation of Retinoblastoma (Rb) protein by CDK4/6 and therefore maintains Rb in the mode of growth-suppression that arrests cells at the G1 phase of the cell cycle [16]. P16^INK4A^ expression gradually increases with aging in most mammalian tissues [17]. The previous study showed P16^INK4A^ accumulated with age and modulated specific functions associated with ageing in stem cells. Increased expression of P16^INK4A^ with age might decrease the self-renewal ability of stem cells [18,19]. In carcinogenic human papillomavirus (HPV) infected cervical cells, HPV E7 oncoprotein inactivates the Rb protein and the E2F family of DNA-binding transcription factors (E2F) release, resulting in the overexpression of P16^INK4A^ [20]. Therefore, P16^INK4A^ is known to be overexpressed in many cervical intra-epithelial neoplasias (CIN) and invasive cervical carcinomas [21,22].

Little is known of the impact of P16^INK4A^ on altering the stem cell markers, cell behaviors of CCSCs, and clinical prognosis of cervical cancer patients who received radiotherapy. In the present study, we aimed to investigate whether patients with a higher expression of P16^INK4A^ in cervical cancer had a significantly better prognosis. Furthermore, we also warranted exploring whether the depleting P16^INK4A^ expression affected the stem cell markers’ expression profiles, self-renewal abilities, radioresistance, and chemoresistance of cervical cancer cells.

## 2. Results

### 2.1. Patient Characteristics

**Figure 1:**
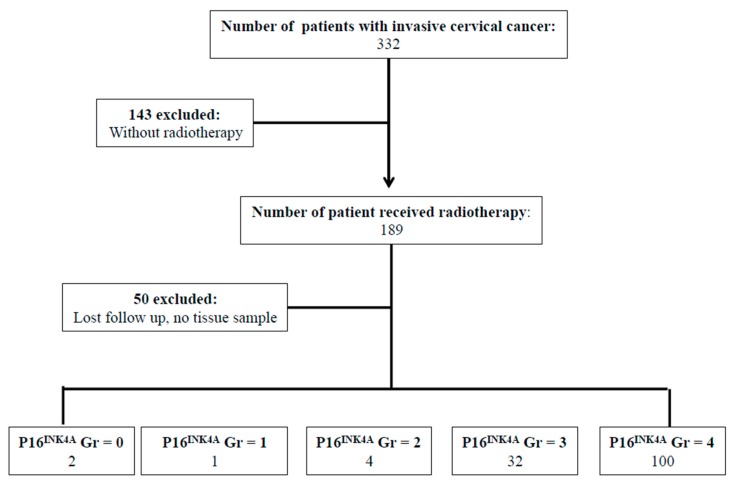
Flowchart of our retrospective study design. Every tumor was given a score according to the extent of stained cells nucleic staining of P16^INK4A^ expression (0% = 0, 1–10% = 1, 11–50% = 2, 51–80% = 3, 81–100% = 4). Abbreviations: Gr: grade.

**Figure 2:**
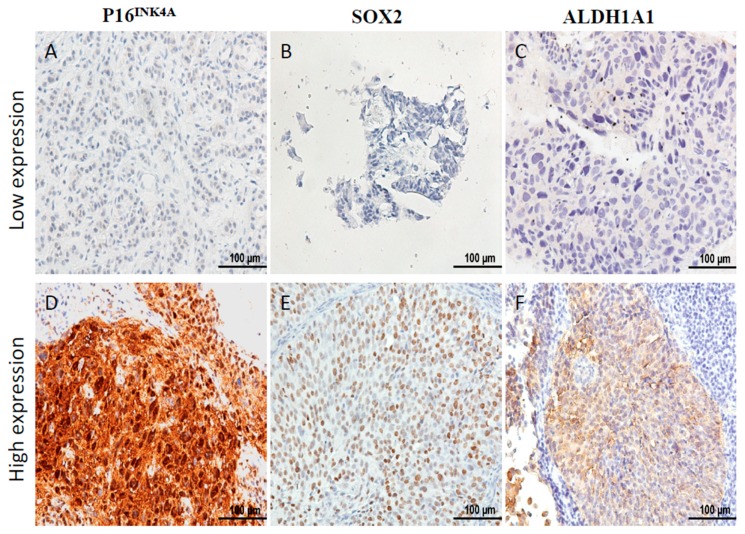
Immunostaining of P16^INK4A^, SOX2, and ALDH1A1 expression in pretreatment cervical cancer. Immunohistochemical staining of P16^INK4A^ expression was low in (**A**) and high in (**D**), SOX2 expression was low in (**B**) and high in (**E**), and ALDH1A1 expression was low in (**C**) and high in (**F**). Scale bar: 100 μm.

Between January 2004 and December 2006, there were 332 patients with pathological proof of cervical cancer in our hospital. We retrospectively reviewed the medical records of the 139 patients that met the criteria of inclusion (Figure 1). To analyze the expression of P16^INK4A^ of the tumor, immunohistochemical (IHC) staining was performed in 139 cervical cancer samples. According to the expression of P16^INK4A^, patients’ tumors were scored with grade 0 (negative), grade 1, grade 2, grade 3, and grade 4 expression of P16^INK4A^, respectively (Figure 1). Next, the expression of P16^INK4A^ detected in ≤80% of patients’ tumors was defined as the low expression group (Figure 2A), and >80% as the high expression group (Figure 2D). Additionally, we also checked the expression of stem cell markers (SOX2 and aldehyde dehydrogenase 1 family, member A1 (ALDH1A1)) in the tumor samples. The expression of SOX2 detected in ≥10% of tumors and ALDH1A1 was detected in ≥50% of tumors were classified as high expression groups (Figure 2E,F, respectively). The median follow-up duration was 73.4 months (range, 2–113 months). A total of 33 patients (23.7%) received hysterectomy and adjuvant radiotherapy and 84 patients (61.8%) were treated with concurrent chemoradiotherapy. During the follow-up period, recurrence of disease and death were observed in 65 and 69 patients, respectively. According to the expression of P16^INK4A^, we separated the patients into low-expression (≤80%) and high-expression (>80%) groups (Table 1). Notably, the rate of recurrence and death were higher in the low-expression group of P16^INK4A^ (*p* = 0.002 and 0.033, respectively). However, we did not find that the clinicopathological variables including age, stage, histologic type, histologic grade, tumor size, squamous cell carcinoma antigen (SCC) level, carcinoembryonic antigen (CEA) level, combined chemotherapy, high expression of SOX2, or high expression of ALDH1A1 displayed a statistically significant difference between the two groups (Table 1).

### 2.2. Survival Pattern of the Patients’ Tumors with Different Expression of P16^INK4A^

**Figure 3:**
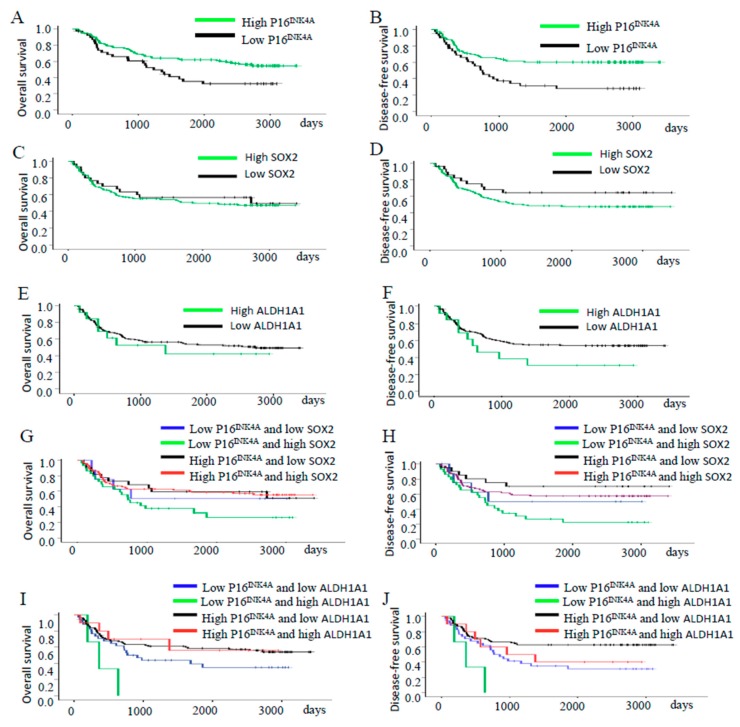
Survival and recurrence outcomes of patients with different expressions of P16^INK4A^, SOX2, and ALDH1A1 in tumors. (**A**,**B**) Cervical cancer patients with high P16^INK4A^ expression had a better five-year OS rate (*p* = 0.016) and better five-year DFS rate (*p* = 0.02) than those with lower expression. (**C**,**D**) Patients with high SOX2 expression had similar five-year OS and DFS than those with low expression (**C**, *p* = 0.598 and **D**, *p* = 0.141). (**E**,**F**) Patients with high ALDH1A1 expression had similar five-year OS and DFS than those with low expression (**E**, *p* = 0.591 and **F**, *p* = 0.131). (**G**,**H**) The patients with low P16^INK4A^/high SOX2 expression had similar five-year OS rates (**G**, *p* = 0.118) but worse five-year DFS rates (**H**, *p* = 0.009) than those with high P16^INK4A^/lower SOX2 expression. (**I**,**J**) The patients with low P16^INK4A^/high ALDH1A1 expression had worse five-year OS rates (**I**, *p* = 0.030) and worse five-year DFS rates (**J**, *p* = 0.003) than those with high P16^INK4A^/lower ALDH1A1 expression.

Next, we separated the patients into different groups according to the expression of the proteins of P16^INK4A^ and stem cell markers SOX2 and ALDH1A1 of the tumor samples obtained before radiotherapy and examined the association among these protein expressions with the five-year overall survival (OS) and disease-free survival (DFS) pattern of the patients. The OS and DFS for the entire cohort were 52.5% and 51.6%, respectively. OS and DFS curves of the patients’ tumors with different expressions of P16^INK4A^ are shown in Figure 3A,B, respectively. The high expression of the P16^INK4A^ group had a higher five-year OS rate and DFS rate than the low expression group (OS: 62.0% and 35.2%, *p* = 0.016; DFS: 60.0% and 31.2%, *p* = 0.002). The high expression of the SOX2 group had similar five-year OS rates and DFS rates to the low expression group (OS: 54.3% and 60.0%, *p* = 0.598; DFS: 48.4% and 64.4%, *p* = 0.141; Figure 3C,D). The high expression of the ALDH1A1 group had similar five-year OS rates and DFS rates to the low expression group (OS: 53.8% and 55.6%, *p* = 0.591; DFS: 30.8% and 54.8%, *p* = 0.131; Figure 3E,F). The patients with low P16^INK4A^/high SOX2 expression had a similar five-year OS rate, but worse five-year DFS rate than those with high P16^INK4A^/lower SOX2 expression (OS: 32.8% and 63.6%, *p* = 0.118; DFS: 26.8% and 70.2%, *p* = 0.009; Figure 3G,H). The patients with a low P16^INK4A^/high ALDH1A1 expression had a worse five-year OS rate and five-year DFS rate than those with high P16^INK4A^/lower ALDH1A1 expression (OS: 0.0% and 61.3%, *p* = 0.030; DFS: 0.0% and 62.7%, *p* = 0.003; Figure 3I,J).

### 2.3. Analysis of Risk Factors for Recurrence of Disease

The results of the univariate (log-rank test) and multivariate (Cox proportional hazard) analyses of DFS are shown in Table 2. Univariate analysis showed advance stage, non-squamous cell carcinoma (SCC) type carcinoma, high SCC antigen level, high carcinoembryonic (CEA) antigen level, low P16^INK4A^ expression, low P16^INK4A^/high SOX2 expression, and low P166^INK4A^/high ALDH1A1 were risk factors of recurrence in patients treated with radiotherapy. Based on the multivariate analysis (Table 2), stage III/IV (hazard ratios (HRs) = 2.950; and 95% confidence intervals (CIs), 1.378–6.317), non-SCC cell type (HRs = 4.770; 95% CIs, 1.597–14.25), and lower expression of P16^INK4A^ (HRs = 1.941; 95% CIs, 1.057–3.559) were significantly poor predictors for DFS.

### 2.4. Depletion of P16^INK4A^ Increased the Resistance to Cisplatin and Irradiation of Cervical Cancer Cells

**Figure 4:**
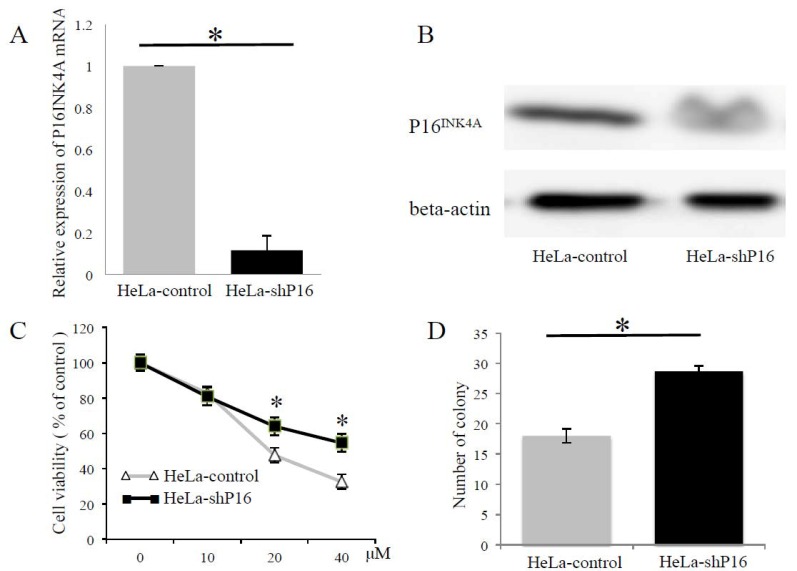
Depletion of P16^INK4A^ increased the resistance to cisplatin and irradiation of cervical cancer cells. (**A**,**B**) P16^INK4A^ expression was down-regulated after pTGMP-p16/p19.478 (HeLa-shP16) transfection. The inhibition of P16^INK4A^ mRNA and protein expression reached 88% and 41%, respectively. Beta-actin was used as a control for RT-PCR and western blotting. Final data were normalized to it. (**C**) Cell viability was evaluated by the CCK-8 assay in different concentrations of cisplatin. (**D**) After ionizing irradiation (6 Gray), cell survival was detected by colony formation assay. The Student’s *t*-test was used for continuous variables between the two groups. * *p* < 0.05. Data are presented as the mean ± SD of three independent experiments.

To further evaluate whether P16^INK4A^ was involved in modulating resistance to cytotoxic agents and irradiation, we knocked-down the expression of P16^INK4A^ by delivering a short hairpin ribonucleic acid (shRNA) specific to P16^INK4A^ in HeLa cells. We selected the stable knockdown cells as HeLa-shP16 cells. The inhibition of P16^INK4A^ mRNA and protein expression reached 88% and 41%, respectively (Figure 4A,B), and the chemo-sensitivity of the HeLa-control and HeLa-shP16 cells was assessed with different concentrations of cisplatin treatments for 24 h by the cell viability counting kit-8 (CCK-8) assay. As shown in Figure 4C, the HeLa-shP16 cells had a higher cell viability at 20 and 40 μM of cisplatin for 24 h of treatment than the HeLa-control cells. Furthermore, we assessed the effect of P16^INK4A^ on the radiation response of cervical cancer cells in vitro. The cell survival efficiency in the HeLa-control and HeLa-shP16 cells was determined by the colony-forming assay (18.0 ± 1.2 vs. 28.6 ± 0.9, *p* < 0.05; Figure 4D). Together, these data demonstrated that the HeLa-shP16 cells exhibited higher survival when compared with the control cells after irradiation.

### 2.5. The Inhibition of P16^INK4A^ Protein Expression Associated with Higher SOX2, ALDH1A1 Expression and Self-Renewal Ability in Cervical Cancer Cells

**Figure 5:**
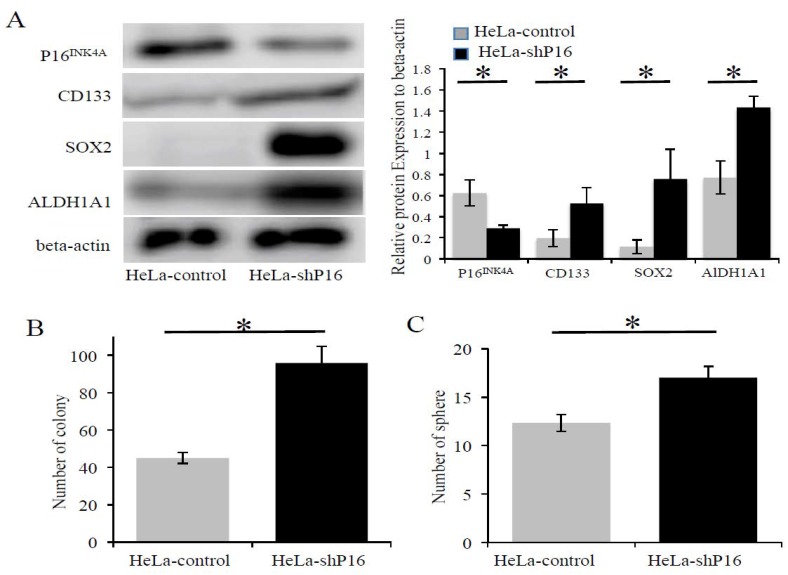
The inhibition of P16^INK4A^ protein expression increased SOX2, ALDH1A1 expression, and self-renewal ability in cervical cancer cells. (**A**) Expression of stem cell markers (CD133, SOX2, and ALDH1A1) at the protein level in the HeLa-control and HeLa-shP16 cells were detected by Western blot and relative protein expression to beta-actin protein was shown. (**B**) Colony formation assay of HeLa-control and HeLa-shP16 cells were used to evaluate the ability of self-renew. (**C**) The sphere formation assay was used to measure the anchorage-independent growth of HeLa-control and HeLa-shP16 cells in ultra-low attachment plates. Each bar represents the mean ± SD of three independent experiments. Student’s *t*-test was used for continuous variables between the two groups. * *p* < 0.05. Data are presented as the mean ± SD of three independent experiments.

Previous studies have shown that the decline in the self-renewal capacity of stem cells with age was partly due to the increased expression of P16^INK4A^ [18,19]. Next, we determined if the stemness properties were increased when the P16^INK4A^ expression was silenced by shRNA. HeLa-shP16 cells had significantly decreased the expression of P16^INK4A^ both at the mRNA (Figure 4A) and protein (Figure 4B and Figure 5A) levels. Furthermore, the inhibition of P16^INK4A^ expression resulted in the higher expression of stem cell-like markers at the protein levels (approximately 1.6-fold for CD-133, 6.2-fold for SOX2, and 1.6-fold for ALDH1A1; Figure 5A). Next, the self-renewal abilities of the cells were investigated by colony formation assay and sphere formation assay. HeLa-shP16 cells had significantly higher colonies than the HeLa-control cells (96.0 ± 4.9 vs. 45.0 ± 1.7, *p* < 0.05; Figure 5B). Moreover, the HeLa-shP16 cells had more tumor spheres when compared to the HeLa-control cells in the sphere formation assay (17.0 ± 1.1 vs. 12.3 ± 0.8, *p* < 0.05; Figure 5C).

## 3. Discussion

In this study, we describe the results of p16^INK4A^ immunohistochemistry for 139 patients with FIGO 2009 stages Ib to IV, treated with radiotherapy, with/without chemotherapy. P16^INK4A^ staining was strongly positive in 100 of the 139 cases examined. Patients with high P16^INK4A^ expression tumors had better OS and DFS than patients with low P16^INK4A^ expression tumors. Previous studies have shown increased P16^INK4A^ expression declined the self-renewal ability of stem cells [18]. Therefore, we checked the stem cell markers (SOX2 and ALDH1A1) of the samples. While we did not find that patients with a high expression of SOX2 or ALDH1A1 tumors had poorer OS and DFS, the patients with low P16^INK4A^/high SOX2 or low P16^INK4A^/high ALDH1A1 expression tumors had a significantly poor prognosis of OS and DFS. To our knowledge, this was the first study to have specifically examined the relationship between P16^INK4A^/SOX2, P16^INK4A^/ALDH1A1 expression, and radiotherapy prognosis in cervical cancer. Our results suggested that cervical cancer patients with high P16^INK4A^ expression tumors had a better prognosis of radiotherapy than patients with low P16^INK4A^ expression tumors.

In agreement with the studies from Lin et al. [23] and Huang et al. [6], which showed that the meta-analysis of patients with high P16^INK4A^ expression cervical cancer had significantly better prognosis, both of these studies and our present study did not find an association between the P16^INK4A^ expression of tumors with stage, tumor size, histologic grade or vascular invasion. Additionally, we did not find an association between P16^INK4A^ expression and other risk factors for cervical cancer such as CEA levels, SCC levels, non-squamous cell carcinoma or without chemotherapy. Moreover, low expression of P16^INK4A^ in the tumor was a significantly poor prognosis factor for OS (HRs = 1.818; 95% CIs, 1.111–2.976) and DFS (HRs = 1.941; 95% CIs, 1.057–3.559) for the patients treated with radiotherapy in our study (Figure 2A,B). Schwarz et al. [24] showed P16 expression was predictive of improved survival outcome after radiotherapy for cervical cancer. The results of the study showed that the five-year specific survival and DFS of the P16^INK4A^-positive group (grades 1–4) vs. P16^INK4A^-negative group were 63% vs. 33% (*p* = 0.07) and 57% vs. 34% (*p =* 0.09), respectively. Survival outcomes were also compared between patients with P16^INK4A^ >50% vs. ≤50% expression. The five-year specific survival and DFS of the high-P16^INK4A^ group vs. the low-P16 group were 64% vs. 24% (*p* = 0.003) and 59% vs. 23% (*p =* 0.0004), respectively. Schwarz et al. used 50% expression of P16^INK4A^ to separate patients into high and low groups and showed a more significant difference in survival outcomes of the two groups. We used the cut-off level of P16^INK4A^ expression as 80% to separate our patients into high and low groups. The patients of low-P16^INK4A^ groups had significantly poor five-year OS (OS: 35.2% vs. 62.0%, *p* = 0.016) and DFS (31.2% vs. 60.0%, *p* = 0.002). Additionally, multivariate analysis showed that lower expression of P16^INK4A^ was one of the independent poor predictors for the DFS of cervical cancer treated with radiotherapy.

Kim et al. [25] reported the expression and clinical significance of cancer stem cell markers (Octamer-binding transcription factor 4 (OCT4) and SOX2) in cervical cancer. In their study, they showed that OCT4 overexpression and loss of SOX2 expression of tumor parts were strongly associated with poor prognosis of patients with cervical cancer [25]. Yao et al. [26] showed that patients with recurrent disease (high vs. low: 88.9% vs. 19.0%) were more likely to have a higher ALDH1 expression. However, Lv et al. [27] had different findings of ALDH1 in cervical cancer patients with chemoradiotherapy. They did not find an association between ALDH1 and survival. We also did not find that the stem cell marker (SOX2 and ALDH1A1) expressions were significantly associated with OS and DFS, although the trend was that those patients with high SOX2 or ALDH1A1 had poorer survival outcomes lacking a statistical difference.

Next, we further investigated whether the P16^INK4A^ expression plus stem cell marker (SOX2 or ALDH1A1) was a good predictor of survival outcomes of cervical cancer patients treated with radiotherapy. Our results showed that patients with low P16^INK4A^/high SOX2 expression had a similar five-year OS rate but worse five-year DFS (Figure 3G,H). Patients with low P16^INK4A^/high ALDH1A1 expression had a worse five-year OS rate and five-year DFS rate (Figure 3I,J).

Li et al. [28] reported that the genetic inhibition of the P16^INK4A^ had an effectively positive effect on the efficiency of induced pluripotent stem cell generation. The data showed that the repression of P16^INK4A^ was found during cells reprogramming and P16^INK4A^ was a barrier of cell reprogramming. Arima et al. [29] showed that a loss of P16^INK4A^ expression was associated with increased stem cells markers and resistance to therapy in breast cancer cells. We inhibited P16^INK4A^ expression in HeLa cells and found a higher expression of stem cell markers (CD133, SOX2, and ALDH1A1, Figure 5A). The cervical cancer cells with lower P16^INK4A^ expression had a higher ability of self-renewal, the essential ability of cancer stem cells (Figure 5B,C). Furthermore, the repression of P16^INK4A^ expression increased the chemoresistance and radioresistance of cells (Figure 4C,D). The results of the cell line were compatible with our clinical findings.

In comparison with other studies [6,21,23,24,30,31] that have shown only either clinical data or cell line data, the strengths of this study were that we only included patients with cervical cancer post radiotherapy with/without chemotherapy, the length of follow-up, the description of recurrence rate, and analysis of the risk factors of recurrence. Moreover, we showed that the cell line data were compatible with the clinical data. The retrospective nature of our current study and the potential for selection bias were the main limitations.

In conclusion, we showed that low expression of P16^INK4A^ was correlated with poor prognosis of the patient with radiotherapy. Patients with low P16^INK4A^ expression with high SOX2 or ALDH1A1 expression had even worse prognosis. We identified advanced stage, non-SCC cervical cancer, and low P16^INK4A^ expression were independent poor factors of DFS. In the cell line data, low P16^INK4A^ expression was associated with a higher expression of stem cell markers and the ability of cells to self-renew. Our data not only provided insights into the role of P16^INK4A^ in cervical cancer, but also suggested that targeting P16^INK4^ expression may act as a novel experimental radiosensitizer with a potential anti-cancer function for developing effective therapeutic strategies against cervical cancer.

## 4. Materials and Methods

### 4.1. Patients and Tissues

This was a retrospective study composed of patients treated between January 2004 and December 2006. Tissue samples from all patients showed pathological proof of cervical cancer. The inclusion criteria were FIGO 2009 stages Ib–IV; the cell types squamous, adenosquamous, or adenocarcinoma; and undergoing radiotherapy, which resulted in 189 patients. After the exclusion criteria of the patients, whose tissues were from outside the hospital, were insufficient, or did not receive regular follow-up, 139 patients were analyzed. All patients underwent external-beam radiotherapy. The details of the radiotherapy have been described previously [5,7]. This study was approved by the Institutional Review Board at Chang Gung Memorial Hospital (CGMH) (number: 106-0344C, 20 February 2017) and conducted in accordance with the approved institutional guidelines. The clinical data were obtained as data released by KCGMH for research purposes. All tissues were obtained from formalin-fixed, paraffin-embedded tissue blocks. Representative areas of tumor were selected by a pathologist from hematoxylin stained sections.

### 4.2. Immunohistochemical Staining

For immunohistochemical staining, we deparaffinized the sections with xylene rinse, rehydrated with a graded alcohol series (100%, 95%, 85%, and 75%), and then rinsed with distilled water. We enhanced antigen retrieval with citrate buffer (10 mM, pH 6.0). Endogenous peroxidase activity was quenched by incubation in a 3% hydrogen peroxide solution. We incubated the slides with primary antibodies, P16^INK4A^ (Roche, Tucson, AZ, USA. Cat#725-4713), SOX-2 (BioSB, Santa Barbara, CA, USA. Cat# BSB 2205), and ALDH1A1 (BioSB Cat# BSB 2443). The slides were further incubated with an indicated secondary antibody. Antigen–antibody complexes were detected by DAB (Dako, Glostrup, Denmark) and counterstained with Gill’s hematoxylin (Merck, Whitehouse, NJ, USA). Staining was graded by two pathologists (I.-C.C. and H.-S.H.) who were blinded to the clinical outcomes and all other data on the patients. Every tumor was given a score according to the extent of stained cells nucleic or cytoplasmic staining of P16^INK4A^ expression (0% = 0, 1–10% = 1, 11–50% = 2, 51–80% = 3, 81–100% = 4; negative means 0% area staining focally positive means 1–80% area staining, diffusely positive means 81–100% area staining) [32]. Expression of SOX2 was graded as 0, less than 10% cells reactive; 1+, 10 to 25% cells reactive; 2+, 26 to 50% cells reactive; 3+, 51 to 75% cells reactive; and 4+, more than 75% cells reactive [33]. Expression of ALDH1A1 was graded as 3+ (≥50% positive tumor cells), 2+ (<50% but ≥10%), 1+ (<10%), or negative (0%) [26].

### 4.3. Cell Culture and Reagents

Cervical cancer cell lines, HeLa cells were obtained from the Bioresource Collection and Research Center (BCRC, Hsinchu, Taiwan) and cell line authentication was performed by BCRC. Cells were cultured in Dulbecco’s modified Eagle’s medium (DMEM) containing 10% fetal bovine serum (FBS), 100 IU/mL penicillin, 100 mg/mL streptomycin, and 0.4 mM l-glutamine (Sigma, St. Louis, MO, USA) in a humidified 95% atmosphere with 5% CO_2_ at 37 °C and passaged for fewer than six months after receipt or resuscitation.

### 4.4. Transfection of Cervical Cancer Cell Lines

The plasmids of pBaBepuro3 and pTGMP-p16/p19.478 were acquired through Addgene. For knockdown expression of P16^INK4A^, the cells were transfected with pTGMP-p16/p19.478. The plasmid of pBaBepuro3 was transfected as control cells. We transfected cells using Lipofectamine 2000 reagent (Life Technologies, Carlsbad, CA, USA) according to the manufacturer’s instructions. After transfection, the cells were grown in puromycin (Sigma, St. Louis, MO, USA) at 5 μg/mL for stable clone selection.

### 4.5. Irradiation of Cells

The 2000 and 1000 HeLa-control and HeLa-shP16 cells were transferred to 25-cm^2^ flasks and incubated in DMEM with 10% FBS at 37 °C with 5% CO_2_ for 24 h. The flasks were placed on a linear accelerator Clinac 600C/D (VARIAN, Palo Alto, CA, USA) with a fixed source skin distance and X-ray irradiation at 4 Gy/min.

### 4.6. Clonogenic Assay

After irradiation (the 2000 and 1000 cells) and before irradiation (100) cells were seeded separately in 25-cm^2^ flasks for colony formation. After 14 days, colonies were fixed and stained with a mixture of 6% glutaraldehyde and 0.5% crystal violet. Only if a single colony contained more than 50 cells was it scored using a microscope [34]. Each assay was performed in duplicate on three independent occasions.

### 4.7. Western Blotting

Cells were lysed in lysis buffer supplemented with 1% protease inhibitor cocktail (Roche Applied Science, Indianapolis, IN, USA). Proteins in whole-cell lysates were resolved by sodium dodecyl sulfate-polyacrylamide gel electrophoresis and transferred to polyvinylidene fluoride (PVDF) membranes. Membranes were probed with anti-P16^INK4A^ (1:1000; Millipore, Burlington, MA, USA, Cat#MAB4133), anti-CD133 antibody (1:1000; Abnova, Taipei, Taiwan,, Cat#PAB12663), anti-SOX2 (1:1000; R&D, Cat#MAB2018), anti-ALDHA1 (1:1000; ThermoFisher, Waltham, MA, USA, Cat#PA5-34901), anti-actin (1:10,000; Millipore, Cat#MAB1501), and then incubated with horseradish peroxidase-conjugated secondary antisera (Amersham, Buckinghamshire, UK). Enhanced chemiluminescence was performed with ECL-Plus (Amersham, Buckinghamshire, UK).

### 4.8. Cellular Toxicity via CCK-8 Assay

The cellular toxicity assay of the HeLa cells was determined using a cell counting kit-8 (CCK-8, Dojindo Molecular Technologies, Rockville, MD, USA) according to the manufacturer’s instructions. In brief, HeLa cells were seeded on a 96-well plate at a density of 3000 cells per well. Then, the cells were treated with increasing concentrations of cisplatin (0–40 μM; Sigma, St. Louis, MO, USA, Cat# 479306) or vehicle (sterile water) for 24 h. CCK-8 was added and the absorbance (optical density, OD) at 450 nm was detected using an enzyme-linked immunosorbent assay reader (Perkin-Elmer; Victor 2V, Waltham, MA, USA).

### 4.9. Sphere Formation Assay

Single cell suspensions were suspended at a density of 2000 cells/mL in DMEM and seeded into ultra-low attachment 6-well plates (Corning Inc., Corning, NY, USA). Suspension cultures were continued for 14 days until tumor-spheres were formed.

### 4.10. Real-Time PCR Assay

Quantitative real time polymerase chain reaction (RT-PCR) was performed using Fast SYBR^TM^ Green Master Mix (ABI, Foster City, CA, USA, Cat# 4385612) and Applied Biosystems^®^ 7500 Fast Real-Time PCR Systems (ABI, Foster City, CA, USA). The cycling parameters for all genes were the following: hot-start 95 °C 15 min, 40 cycles of (denaturation 95 °C 3 s, annealing 60 °C 30 s, elongation 72 °C 30 s, plate read). The PCR primer sequences used are as follows: P16^INK4A^ Forward primer 5′CCC ACC GCA CCG AAT AGT TA3′, Reverse primer 5′ACC AGC GTG TCC AGG AAG3′; OCT-4 Forward primer 5′CCA CAT CGG CCT GTG TAT ATC3′, Reverse primer 5′AGC AAA ACC CGG AGG AGT3′; CD133 Forward primer 5′CAA CCC TGA ACT GAG GCA GC3′, Reverse primer 5′TTG ATA GCC CTG TTG GAC CAG3′; SOX2 Forward primer 5′GGG AAA TGG GAG GGG TGC AAA AGA GG3′, Reverse primer 5′TTG CGT GAG TGT GGA TGG GAT TGG TG3′; ALDH1 Forward primer 5′CTG CTG GCG ACA ATG GAG T3′, Reverse primer 5′GTC AGC CCA ACC TGC ACA G3′; beta-actin Forward primer 5′TCA CCC ACA CTG TGC CCA TCT ACG3′, Reverse primer 5′ CAG CGG AAC CGC TCA TTG CCA ATG3′. We used beta-actin as a control and final data were normalized to it.

### 4.11. Statistical Analysis

Comparisons between the P16^INK4A^ high-expression (≤80%) and low-expression (>80%) groups for clinicopathological factors were evaluated using the *x*^2^ with Fisher exact for categorical variables and the Student’s *t*-test was used for continuous variables. The median follow-up duration was 73.4 months (range, 2–113 months) for 139 independently validated cases. The endpoint analyzed was disease-free survival (DFS), last visit, or death (overall survival, (OS)). The durations were calculated from the date of operation until the occurrence of recurrence, death, or last follow-up appointment. Univariate survival analyses were conducted using Kaplan–Meier plots and the statistical significance of the difference between the curves were evaluated by the two-sided log-rank test. Furthermore, hazard ratios (HRs) and 95% confidence intervals (CIs), which were computed from univariate and multivariable Cox proportional hazards regression models were used to assess associations between the higher and lower expression of P16, recurrence, and survival. All statistical analyses were performed using the SPSS statistical package. All *p* values less than 0.05 were considered statistically significant.

## Figures and Tables

**Table 1:** Patient characteristics.

Variable	Total (%)	Status of P16^INK4A^ Staining		*p* Value
		Low	High	
All cases	139	39	100	
Age				
≥60	69 (49.6)	21 (53.8)	48 (48.0)	
<60	70 (50.4)	18 (46.2)	52 (52.0)	0.536
Stage				
lower (I,II)	110 (79.1)	29 (74.4)	81 (81.0)	
higher (III,IV)	29 (20.9)	10 (25.6)	19 (19.0)	0.387
Histologic type				
squamous cell carcinoma	131 (94.2)	35 (89.7)	96 (96.0)	
adenocarcinoma	3 (2.2)	2 (5.1)	1 (1.0)	
adenosquamous cell carcinoma	5 (3.6)	2 (5.1)	3 (3.0)	0.261
Histologic grade				
Grade 1	10 (7.2)	2 (5.1)	8 (8.0)	
Grade 2	36 (25.9)	10 (25.6)	26 (26.0)	
Grade 3 and unknown	93 (66.9)	27 (69.2)	66 (66.0)	0.832
Tumor size				
≥4 cm	77 (55.4)	21 (53.8)	56 (56.0)	
<4 cm	62 (44.6)	18 (46.2)	44 (44.0)	0.818
SCC level before therapy (SD)	12.5 (26.9)	14.3 (24.6)	12.2 (28.8)	0.703
CEA level before therapy (SD)	14.2 (57.4)	20.6 (62.3)	12.6 (58.2)	0.527
High SOX2 #	107 (77.5)	29 (76.3)	78 (78.0)	0.832
High ALDH1A1 ##	13 (9.6)	3 (7.9)	10 (10.0)	0.681
Hysterectomy	33 (23.7)	11 (28.2)	22 (22.0)	0.440
Chemotherapy	84 (61.8)	24 (61.5)	60 (60.0)	0.998
Recurrence	65 (47.8)	27 (69.2)	38 (38.0)	0.002 *
Deaths	69 (49.6)	25 (64.1)	44 (44.0)	0.033 *

Continuous data are presented as the mean ± standard deviation, # ≥10%, ## ≥50%, * *p* < 0.05. Abbreviations: SD, standard deviation; SCC, squamous cell carcinoma antigen; CEA, carcinoembryonic antigen.

**Table 2:** Univariate and multivariate logistic regression analysis of the prognostic factors for recurrence.

Variable	Univariate Analysis			Multivariate Analysis		
	HR	95% CI	*p* Value	HR	95% CI	*p* Value
Age ≥ 60	1.043	0.652–1.668	0.86	1.348	0.702–2.591	0.369
Stage (III, IV)	3.607	2.159–6.026	<0.001 *	2.95	1.378–6.317	0.005 *
Cell type (non-SCC)	3.009	1.489–6.080	0.002 *	4.77	1.597–14.25	0.005 *
Histologic grade (G3)	1.42	0.928–2.171	0.106	1.306	0.763–2.237	0.33
Tumor size (≥4 cm)	1.587	0.973–2.591	0.064	1.053	0.518–2.141	0.888
High SCC level #	1.845	11.06–3.078	0.019 *	1.312	0.640–2.687	0.459
High CEA level ##	3.134	1.607–6.110	0.001 *	2.077	0.931–4.632	0.074
Low P16^INK4A^	2.137	1.302–3.509	0.003 *	1.941	1.057–3.559	0.032 *
Low P16^INK4A^/high SOX2	2.298	1.369–3.857	0.002 *	0.85	0.160–4.509	0.849
Low P16^INK4A^/high ALDH1A1	4.086	1.261–13.24	0.019 *	1.608	0.276–9.381	0.598

# ≥ 5 ng/mL, ## ≥ 10 ng/mL, * *p* < 0.05. Abbreviations: HR, hazard ratio; CI, confidence interval; SCC, squamous cell carcinoma; CEA, carcinoembryonic antigen.
